# UK Biobank-Based Genetic and Proteomic Network Insights into Metabolic Dysfunction-Associated Steatotic Liver Disease Pathogenesis

**DOI:** 10.3390/ijms27093920

**Published:** 2026-04-28

**Authors:** Sang Wook Kang, Su Kang Kim, Ju Yeon Ban, Min Su Park

**Affiliations:** 1 Department of Oral and Maxillofacial Pathology, College of Dentistry, Kyung Hee University, Seoul 02447, Republic of Korea; 2 Department of Biomedical Laboratory Science, Catholic Kwandong University, Gangneung 25601, Republic of Korea; 3 Department of Dental Pharmacology, College of Dentistry, Dankook University, Cheonan 31116, Republic of Korea; 4 Department of Surgery, School of Medicine, Kyung Hee University, Seoul 02447, Republic of Korea

**Keywords:** metabolic dysfunction-associated steatotic liver disease, proteomics, protein–protein interaction (PPI) network, genome-wide association study, fatty liver, nonalcoholic, metabolic syndrome

## Abstract

Metabolic dysfunction-associated steatotic liver disease (MASLD) is increasingly recognized as a systemic disorder shaped by genetic variants and network-level interactions beyond obesity and insulin resistance. This study aimed to define the genetic and proteomic architecture of MASLD by integrating GWAS and plasma proteomic profiling from the UK Biobank. Genome-wide association analyses were conducted under additive and dominant models, with functional annotations performed using SIFT, PolyPhen-2, PROVEAN, REVEL, CADD, MutationTaster, and conservation metrics (GERP++, phyloP, phastCons, and B-statistic). Differential protein expression was assessed using the Olink^®^ platform, and STRING was applied for protein–protein interaction analysis. MASLD patients showed male predominance and significant differences in hepatic (AST, ALT, GGT, PDFF), metabolic (glucose, triglycerides, TyG index), and inflammatory markers (CRP, neutrophils, NLR, CAR). GWAS confirmed *PNPLA3* (rs738409, I148M) and *TM6SF2* (rs58542926, E167K) as major risk variants, while *SAMM50* and *NCAN* showed weaker but conserved associations. Proteomics revealed downregulation of IGFBP2, IGFBP1, PON3, CKB, and APOF and upregulation of CPM, IGSF9, GUSB, ACY1, AFM, LEP, and GSTA1/3. PPI analysis identified ADIPOQ, LEP, FGF21, and ADH1B as central hubs in metabolic and inflammatory regulation. MASLD should be regarded as a network disease involving lipid metabolism, insulin/IGF signaling, mitochondrial function, and ECM–inflammatory pathways. These findings highlight *PNPLA3* and *TM6SF2* as major genetic drivers, while *SAMM50*, *NCAN*, and peripheral proteins contribute regulatory roles, suggesting novel biomarkers and therapeutic targets.

## 1. Introduction

Non-alcoholic fatty liver disease (NAFLD) has traditionally been used to describe the accumulation of fat in the liver in the absence of significant alcohol consumption. However, this terminology has several limitations, including its narrow, exclusion-based definition and the potentially stigmatizing connotations of the terms “non-alcoholic” and “fatty.” To address these issues, the term metabolic dysfunction-associated steatotic liver disease (MASLD) has recently been proposed, providing a more accurate and etiologically relevant description. This updated terminology emphasizes that hepatic steatosis should be interpreted in the context of metabolic dysfunction and its strong associations with conditions such as obesity, diabetes, and hypertension [1].

MASLD is not merely a pathological phenomenon confined to the liver; rather, it is closely linked to a broad spectrum of systemic diseases, including metabolic syndrome, type 2 DM, cardiovascular disease, chronic kidney disease, and even extrahepatic malignancies [2,3]. These associations underscore that MASLD is a systemic metabolic disorder rather than an isolated hepatic condition. Previous studies have demonstrated that patients with MASLD have a significantly increased risk of atherosclerosis, heart failure, and arrhythmias, and that MASLD shares overlapping pathophysiological pathways with insulin resistance and dyslipidemia, positioning it as an important component of the cardiometabolic disease spectrum [4,5]. While obesity, insulin resistance, and dyslipidemia are well-established drivers of MASLD, emerging evidence indicates that gut microbiota dysbiosis, environmental exposures, sedentary lifestyle, and certain medications further contribute to its complex pathogenesis [6,7].

Beyond metabolic and environmental influences, genetic factors play a pivotal role in MASLD susceptibility and disease progression [8]. Twin and family studies have consistently demonstrated the heritability of hepatic fat content, suggesting a strong genetic predisposition [9,10]. Several candidate gene variants—including *PNPLA3 (I148M)*, *TM6SF2 (E167K)*, *MBOAT7*, and *HSD17B13*—have been repeatedly identified as major genetic determinants affecting hepatic fat accumulation, inflammation, and fibrosis risk [11,12,13,14]. These findings underline the importance of genetic exploration not only for understanding disease heterogeneity but also for improving risk stratification and advancing biological understanding of MASLD.

In this context, the UK Biobank, a prospective cohort of over 500,000 participants, provides a unique resource for MASLD research [15]. By integrating genetic, clinical, biochemical, and lifestyle data, it supports a comprehensive investigation of gene–environment interactions in metabolic liver disease. In this study, we leveraged UK Biobank-based GWAS and plasma proteomic profiling to characterize genetic association signals in MASLD within a stringent case–control framework and to explore circulating molecular signatures related to metabolic and cardiovascular traits. As a population-based resource, it provides a valuable framework for investigating genetic and circulating molecular features of MASLD, thereby helping to inform future precision medicine approaches. Whereas GWAS identifies inherited susceptibility signals, plasma proteomics may reflect downstream or parallel systemic alterations associated with MASLD.

Therefore, the purpose of this study was to investigate genetic association signals and plasma proteomic features associated with MASLD, thereby deepening our understanding of the disease’s pathophysiology and identifying biologically relevant pathways for future study.

## 2. Results

### 2.1. Characteristics of Participants

Table 1 shows the characteristics of participants. In the case group, the proportion of males was higher than that of females. The measurements of proton density fat fraction (PDFF), glucose, triglycerides (TG), aspartate aminotransferase (AST), alanine aminotransferase (ALT), gamma-glutamyl transferase (GGT), c-reactive protein (CRP), neutrophil count, lymphocyte count, monocyte count, low-density lipoprotein (LDL), triglyceride-glucose (TyG) index, neutrophil-to-lymphocyte ratio (NLR), prognostic nutritional index (PNI), and c-reactive protein-to-albumin ratio (CAR) indicated statistically significant differences between the case group and the control group. In contrast, body mass index (BMI), high-density lipoprotein (HDL), diastolic blood pressure (DBP), systolic blood pressure (SBP), platelet count, albumin, lipoprotein, total protein, platelet-to-lymphocyte ratio (PLR), neutrophil-to-monocyte ratio (NMR), and albumin-to-globulin ratio (AGR), as well as the prevalence of diabetes mellitus (DM), hypertension (HTN), and hyperlipidemia, did not differ significantly between the two groups.

### 2.2. Genome-Wide Association Analyses Were Performed Under Additive and Dominant Genetic Models

**Figure 1:**
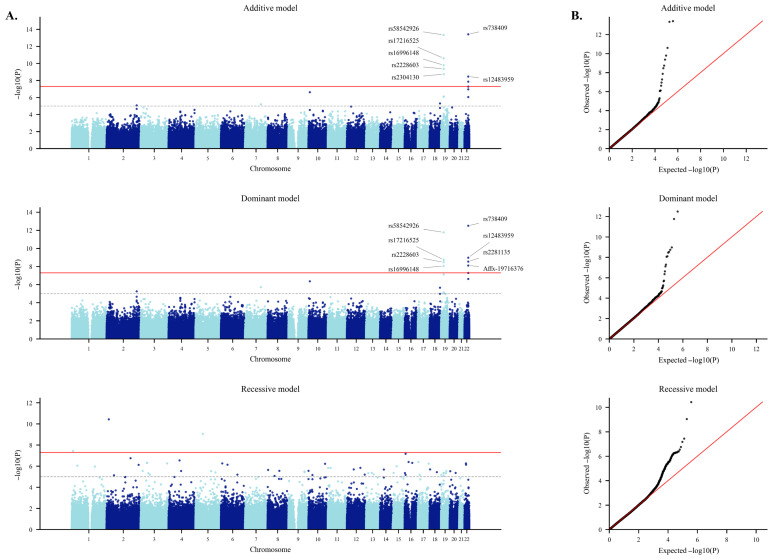
Manhattan plots and quantile–quantile plots of genome-wide association analysis under three genetic models. (**A**) Manhattan plots showing genome-wide associations under the additive (**top**), dominant (**middle**), and recessive (**bottom**) genetic models. Each point represents a single nucleotide polymorphism (SNP), plotted according to chromosomal position on the *x*-axis and –log_10_(*p*-value) on the *y*-axis. Alternating colors distinguish adjacent chromosomes for visual clarity. The horizontal red dashed line indicates the genome-wide significance threshold (*p* = 5 × 10^−8^). (**B**) QQ plots comparing observed versus expected −log_10_(*p*-values) under the additive (**top**), dominant (**middle**), and recessive (**bottom**) models. The red dashed line represents the null distribution. The observed values closely follow the expected distribution, with deviation observed in the upper tail, suggesting the presence of true association signals with minimal evidence of genomic inflation.

Table 2 shows the results of the logistic regression analysis. Under the additive model, multiple loci reached genome-wide significance (*p* < 5 × 10^−8^). The most significant associations were observed at rs738409 in *PNPLA3* (Chr22, I148M, *p* = 3.77 × 10^−14^, OR = 1.56) and rs58542926 in *TM6SF2* (Chr19, E167K, *p* = 4.51 × 10^−14^, OR = 1.92). Additional significant variants were detected within the *TM6SF2* locus (rs17216525, rs16996148, rs2228603) and in nearby regions such as *ZNF101* (rs2304130). Several intronic variants within *PNPLA3* (rs12483959, rs2281135) also showed strong signals, consistent with extended linkage disequilibrium (LD) around the lead coding variant. Figure 1 presents Manhattan and quantile–quantile plots under additive, dominant, and recessive genetic models. However, results from the recessive model were considered exploratory due to limited statistical stability and were not included in the main interpretation. The QQ plot for the additive model demonstrated clear deviation from the null distribution, confirming robust association signals with minimal evidence of inflation. In the dominant model, the strongest signals again involved rs738409 (PNPLA3, *p* = 3.21 × 10^−13^, OR = 1.69) and rs58542926 (TM6SF2, *p* = 1.74 × 10^−12^, OR = 1.91). Additional significant associations were observed in the PNPLA3 intronic region (rs12483959, rs2281135, Affx-19716376) and in the TM6SF2 region (rs17216525, rs2228603, rs16996148), along with a coding variant in SAMM50 (rs3761472, A110G, *p* = 5.10 × 10^−8^). These findings highlight the consistency of signals across additive and dominant inheritance assumptions. The QQ plot showed modest upward deviation at the tail, again suggesting true associations with limited genomic inflation.

### 2.3. Functional Prediction Results of Major Genetic Variants Associated with MASLD

Table 3 presents the functional prediction results for genetic variants identified as significantly associated with MASLD across multiple predictive algorithms. rs738409 (*PNPLA3*, I148M) was consistently predicted as a damaging variant by SIFT, PolyPhen-2, MutationTaster, PROVEAN, and REVEL, supporting its role as the strongest genetic risk factor for MASLD. rs58542926 (*TM6SF2*, E167K) scored highly on PolyPhen-2 and CADD, suggesting a high probability of functional impairment. rs2228603 (*NCAN*, P → S) is predicted to be potentially damaging based on PolyPhen-2 and CADD. rs3761472 (*SAMM50*, D → G) is classified as benign/neutral or a polymorphism by most algorithms. Therefore, its pathogenic impact appears to be limited compared to other variants.

### 2.4. Conservation Analysis of MASLD-Associated SNPs

Table 4 shows the conservation analysis of MASLD-associated SNPs. rs738409 in *PNPLA3*, although showing strong statistical significance in logistic regression analysis and functional prediction as a canonical MASLD variant, exhibited relatively low conservation scores, while its B-statistic was high, suggesting constraint in human populations [16]. rs58542926 in *TM6SF2* demonstrated very high conservation and functional scores, supporting its role as a major causal variant in MASLD. rs3761472 in *SAMM50* showed strong evolutionary conservation but was predicted to be benign by in silico tools, indicating a potentially subtle regulatory effect. rs2228603 in *NCAN* also displayed high conservation, suggesting that this variant may exert a functional impact relevant to MASLD pathogenesis.

### 2.5. Differential Protein Expression Profiles Between MASLD and Control Group

**Figure 2:**
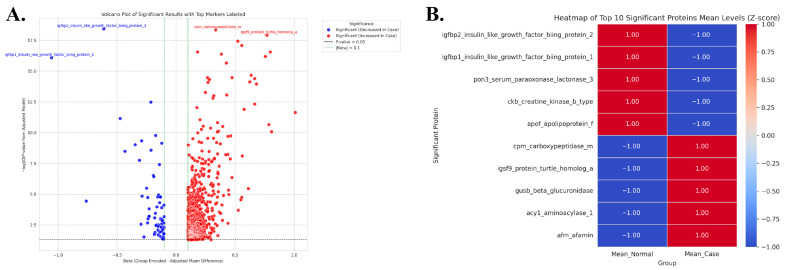
Volcano plot and heatmap of differentially expressed plasma proteins associated with proton density fat fraction. (**A**) Volcano plot showing significantly upregulated (red) and downregulated (blue) plasma proteins associated with hepatic proton density fat fraction (PDFF). The *x*-axis shows the effect size (coefficient), and the *y*-axis shows −log_10_ of the adjusted *p*-value. Labeled proteins passed the significance threshold (adjusted *p* < 0.05). (**B**) Heatmap showing the top 5 upregulated and top 5 downregulated proteins associated with hepatic PDFF. Colors represent normalized expression levels (z-scores), with red indicating high and blue indicating low expression.

Table 5 and Figure 2 present the differential protein expression profiles in MASLD and normal groups. The volcano plot in Figure 2A illustrates proteins with significant expression differences between the MASLD group (case) and the normal group (control) based on protein expression analysis. In particular, IGFBP2 and IGFBP1 were more highly expressed in the normal group and downregulated in the MASLD group (left, blue). Conversely, CPM and IGSF9 showed increased expression in the MASLD group (right, red). These findings highlight a distinct expression pattern characterized by reduced levels of growth factor–binding proteins and elevated levels of inflammation-related proteins in MASLD. As shown in the heatmap of the top 10 significant proteins (Figure 2B), two distinct clusters were evident: proteins highly expressed in the normal group (IGFBP1, IGFBP2, PON3, CKB, APOF) and proteins highly expressed in the MASLD group (CPM, IGSF9, GUSB, ACY1, AFM). In the normal group, proteins involved in growth regulation and lipid metabolism stability were predominant, whereas in the MASLD group, proteins related to protein degradation and inflammatory responses were enriched. Table 5 presents the quantitative values corresponding to Figure 2A,B. Proteins such as IGFBP2, IGFBP1, PON3, CKB, and APOF were highly expressed in the normal group but decreased in the MASLD group (decreased in MASLD, blue in the volcano plot, left side of the heatmap). In contrast, CPM, IGSF9, GUSB, ACY1, and AFM were upregulated in the MASLD group and downregulated in the normal group (increased in MASLD, red in the volcano plot, and on the right side of the heatmap).

### 2.6. PPI Network Construction and Cluster Analysis

**Figure 3:**
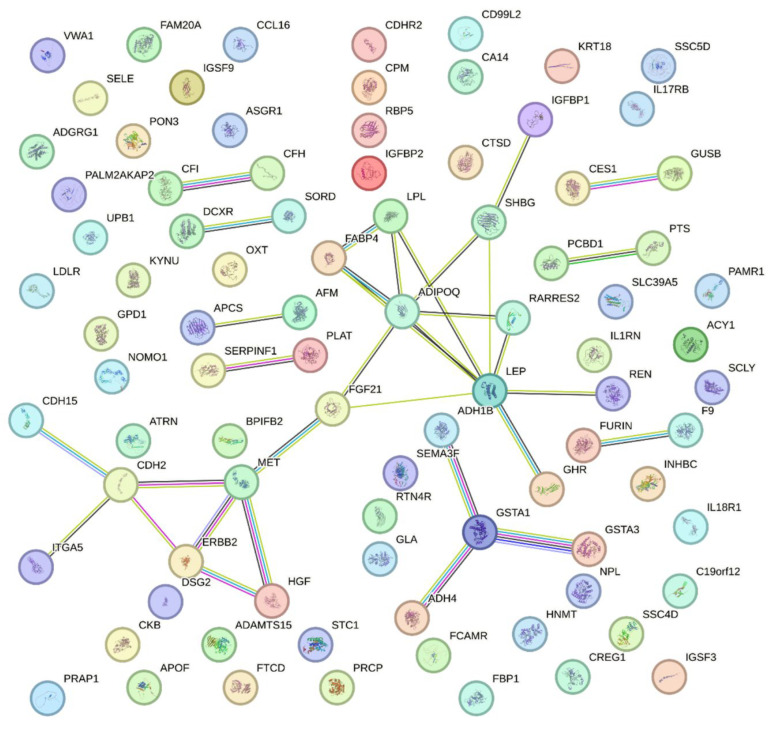
PPI network of differentially expressed plasma proteins associated with proton density fat fraction. The network was constructed using STRING with the following parameters: only query proteins (first shell only) were included, and edges represent evidence-based interactions from active sources including text mining, experimental data, databases, and co-expression. A minimum required interaction score of 0.700 (high confidence) was applied. Colored edges represent different types of interaction evidence. The resulting network highlights key protein hubs such as ADIPOQ, LEP, FABP4, and GSTA1, which may play central roles in the regulation of hepatic fat accumulation.

A protein–protein interaction network was constructed using the STRING database. A total of 15 clusters were identified through MCL clustering (inflation = 2.0), and the number of proteins as well as the functional characteristics of each cluster are summarized in Table 6. These clusters can be broadly categorized as metabolism/hormone-related (clusters 1, 4, 5, 6, and 9), immunity/inflammation-related (clusters 3, 11, and 14), lipid/lipoprotein-related (cluster 7), and others. As shown in Figure 3, cAMP-related genes (Cluster 1) are positioned at the center of the network, whereas the complement pathway (Cluster 3), LDL metabolism (Cluster 7), drug metabolism (Cluster 5), and the IL-1 receptor pathway (Cluster 11) are distributed at the periphery.

## 3. Discussion

In this study, we compared MASLD patients with a control group and comprehensively analyzed clinical indicators, genetic variants, protein expression patterns, and network-level connectivity. As shown in Table 1, consistent with previous reports, the prevalence of MASLD was higher in males [17]. Significant differences were observed in liver-related parameters, including AST, ALT, GGT, and PDFF, which directly reflects hepatic fat content. Moreover, notable differences were found in metabolic markers (glucose, TG, TyG index) and inflammatory/immune-related markers (CRP, neutrophils, lymphocytes, monocytes, NLR, and CAR). In contrast, no significant differences were detected in clinical diagnostic variables such as blood pressure or diabetes status. These findings suggest that MASLD manifests along a continuum of metabolic imbalance and inflammatory responses, rather than as a discrete disease entity like hypertension or diabetes.

Genetic analysis identified rs738409 (*PNPLA3*, I148M) and rs58542926 (*TM6SF2*, E167K) as the strongest MASLD-associated variants, consistent with previous reports [11,12]. Their functional significance was further supported by protein prediction and conservation analyses. Across multiple tools (SIFT, PolyPhen, REVEL, and CADD), *PNPLA3* and *TM6SF2* were consistently classified as damaging variants, whereas SNPs in *NCAN* and *SAMM50* were largely predicted to be benign. Interestingly, despite low interspecies conservation, *PNPLA3* showed a high B-statistic within humans, suggesting that variation at this locus is selectively constrained and functionally important. The B-statistic thus complements interspecies conservation metrics (GERP++, phyloP, phastCons) by highlighting regions under strong constraint in the human population. *TM6SF2* lies within a highly conserved region, and mutations were predicted to strongly impact lipid metabolism, consistent with its established role in MASLD pathogenesis. By contrast, rs3761472 in *SAMM50* and rs2228603 in *NCAN* showed limited functional predictions, but their recurrent associations in GWAS and conservation analyses suggest indirect contributions at the pathway level. *SAMM50* plays a critical role in mitochondrial protein assembly and cristae organization, and its dysfunction leads to impaired oxidative phosphorylation and fatty acid β-oxidation. This disruption might promote hepatic lipid accumulation, increase oxidative stress, and alter energy metabolism and inflammatory responses in MASLD [18]. The *NCAN* rs2228603 variant has been known to be associated with increased hepatic steatosis, inflammation, and perivenular fibrosis in morbidly obese patients, despite paradoxically lower circulating lipid levels [19].

Olink-based protein expression analysis revealed distinct differences between MASLD patients and controls. In the volcano plot and heatmap, IGFBP2, IGFBP1, PON3, CKB, and APOF were elevated in the normal group but suppressed in MASLD, whereas CPM, IGSF9, GUSB, ACY1, AFM, LEP, PRAP1, GSTA1/3, ADH4, GHR, FABP4, and INHBC were upregulated in MASLD. Proteins enriched in the normal group are associated with growth factor binding [20,21], antioxidant defense [22,23], and lipid metabolism [24], while those elevated in MASLD are linked to protein degradation [25,26], oxidative stress [27,28], inflammatory response [29,30,31,32,33], and fatty acid binding/transport [34,35]. These findings suggest that MASLD pathophysiology extends beyond simple fat accumulation and reflects a broader disturbance in metabolic and immune regulation.

The major hub proteins identified in the PPI analysis (Table 6 and Figure 3) were ADIPOQ, FGF21, LEP, IGFBP1/2, LPL, GHR, FABP4, SELE, and LDLR, which were either clustered at the network’s core or acted as bridges connecting central and peripheral modules. These proteins are highly likely to function as endocrine regulators integrating energy metabolism with immune regulation, thereby constituting a key axis in the metabolism–inflammation crosstalk [36,37]. These hub proteins are functionally associated with key biological pathways, including insulin signaling, adipokine regulation, lipid metabolism, and inflammatory responses. ADIPOQ and LEP play central roles in adipokine-mediated metabolic regulation, linking adipose dysfunction to insulin resistance and systemic inflammation, whereas FGF21 is involved in hepatic lipid metabolism and energy homeostasis and may reflect a compensatory response to hepatic metabolic stress. Collectively, these findings suggest that MASLD is associated not only with altered circulating protein levels, but also with coordinated disruption of metabolic and inflammatory signaling networks. Viewed together, these proteins support a mechanistic framework in which dysregulated adipokine signaling, impaired metabolic homeostasis, and inflammatory activation jointly contribute to MASLD pathophysiology. However, it should be noted that formal pathway enrichment analyses (e.g., GO or KEGG) were not performed in this study, which may limit the depth of functional interpretation. Future studies incorporating systematic enrichment approaches will be necessary to further define the biological organization of these protein networks.

The enrichment of many cAMP-responsive proteins as hubs in Cluster 1 further supports the notion that cAMP is more than a mere energy signaling molecule—it may act as a hub signal coordinating metabolic and immune-inflammatory responses [38,39]. In the normal group, IGFBP1/2, PON3, and APOF (which were highly expressed) occupy central or bridging roles within lipid metabolism and insulin-sensitivity regulatory pathways, reflecting a state of systemic metabolic homeostasis and protective lipid handling. In contrast, proteins upregulated in the MASLD group—AFM, GUSB, ACY1, CPM—belong to pathways of complement activation, catabolic/metabolic reprogramming, and inflammatory networks, indicating a shift toward immune–metabolic imbalance and inflammatory remodeling in disease conditions.

However, it should be noted that the PPI network was constructed using only differentially expressed proteins, which may introduce bias toward proteins that are more abundant, better annotated, or more extensively studied in existing interaction databases. As a result, less abundant or less-characterized proteins may be underrepresented, and the resulting network may not fully reflect the complete interaction landscape underlying MASLD. Therefore, the PPI analysis should be interpreted as a focused representation of interactions among significantly altered proteins rather than a comprehensive network model.

Thus, while the normal group displays a dominant protective metabolic/lipid network, the MASLD group shows a predominance of inflammation- and catabolism-related networks. Many of the hub proteins are secreted factors, which raises their potential as blood-based biomarkers. The central hub proteins in the normal group (IGFBP1/2, PON3, APOF) could serve as prophylactic or diagnostic biomarkers reflecting protective metabolic states, whereas the overexpressed proteins in MASLD (AFM, CPM, GUSB, ACY1) may act as prognostic indicators reflective of disease progression and inflammation. Moreover, hub proteins such as ADIPOQ, FGF21, and LEP hold promise as therapeutic targets, offering novel avenues for MASLD treatment strategy development.

Synthesizing the GWAS findings with PPI results, we found that although direct gene–protein one-to-one correspondence was not evident, convergent links emerged at the pathway level. *PNPLA3* is strongly associated with hepatic fat accumulation and modulation of the insulin/IGF signaling axis [40], while *TM6SF2* may influence lipoprotein metabolism via effects on VLDL secretion, thereby impacting downstream proteins such as APOF and PON3 [41,42,43,44]. *SAMM50*, a mitochondrial outer membrane protein, appears to contribute to mitochondrial dysfunction, possibly triggering proteolytic degradation pathways and oxidative stress [45,46,47]. *NCAN*, as an extracellular ECM proteoglycan, may modulate inflammatory responses and structural remodeling, possibly influencing proteins such as IGSF9 and CPM [48,49].

Several limitations should be acknowledged. First, although cases and controls were defined to reflect the MASLD framework using hepatic steatosis and metabolic abnormalities, alcohol intake and other chronic liver diseases were not applied as strict exclusion criteria. Therefore, some degree of residual heterogeneity or misclassification may remain. Second, the case definition was based on an operational classification using imaging-derived PDFF and metabolic status within the UK Biobank dataset, rather than on a fully adjudicated clinical diagnosis of MASLD. Accordingly, the findings should be interpreted with caution when generalizing to clinically defined MASLD populations.

Furthermore, the lack of significant differences in BMI, blood pressure, and diabetes status between groups may reflect the characteristics of the study population, which was drawn from a subset with available proteomic data and limited metabolic stratification. This relatively homogeneous metabolic profile may have attenuated differences in conventional clinical parameters while enabling the identification of proteomic changes associated with hepatic steatosis. Therefore, the findings should be interpreted with caution when generalizing to more clinically stratified MASLD populations.

Additionally, multiple SNPs identified within the same genomic loci were not subjected to fine-mapping or conditional analyses, which limits the ability to distinguish independent association signals and to pinpoint causal variants. Given that variants within the same locus are often in linkage disequilibrium, the present study focused on locus- and pathway-level interpretation rather than variant-level inference. Future studies incorporating fine-mapping and conditional analyses will be required to resolve these associations at higher resolution and to identify causal variants.

Moreover, the proteomic data used in this study were derived from plasma, which may not fully reflect intrahepatic molecular processes. Circulating protein levels represent systemic responses and may differ from liver-specific expression patterns. Accordingly, the identified proteomic signatures should be interpreted as reflecting systemic alterations associated with MASLD rather than liver-specific mechanisms. Future studies incorporating liver tissue–based analyses will be necessary to validate the biological relevance of these findings and to clarify tissue-specific effects.

In addition, the integration between genetic variants and protein expression remained limited, as direct gene–protein correspondence was not consistently observed. This reflects the complexity of multi-layer biological regulation, where genetic effects may be mediated through intermediate molecular processes. Future studies incorporating integrative approaches such as expression quantitative trait loci (eQTL) or mediation analyses will be necessary to better elucidate these relationships.

Furthermore, the PPI analysis was primarily based on hub protein identification and qualitative interpretation. The lack of quantitative network metrics (e.g., degree or centrality measures) and formal pathway enrichment analyses (e.g., GO or KEGG) may limit the depth of functional interpretation. Future studies incorporating these approaches will be necessary to further elucidate the biological organization of the network.

Therefore, the findings should be interpreted with caution when generalizing to clinically defined MASLD populations.

This study suggests that MASLD is not merely a fat storage disorder but a complex network disease involving both metabolic and immune dysregulation. *PNPLA3* and *TM6SF2*, well-established genetic variants from GWAS, were reaffirmed as the strongest genetic risk factors in this analysis. Although evidence for *SAMM50* and *NCAN* has been less consistent, network-based analysis indicated possible functional associations. Collectively, these variants are connected to lipid metabolism, the insulin/IGF axis, mitochondrial function, and ECM-inflammatory responses, implying a multifaceted contribution to MASLD pathophysiology. Protein expression analysis further revealed clear differences between normal and MASLD groups: proteins related to growth regulation and lipid stability were downregulated, whereas those involved in inflammation and oxidative stress were upregulated. Notably, IGFBP2, CPM, ADIPOQ, and LEP emerged as central nodes in the network, suggesting potential utility as diagnostic or prognostic biomarkers, in conjunction with GWAS-identified variants. Nonetheless, direct one-to-one correspondence between genetic variants and protein expression remained limited, reflecting the complexity of multi-layer biological regulation, in which genetic effects are mediated through intermediate molecular processes rather than direct gene–protein relationships. While our findings provide insight at the pathway and network levels, further integrative analyses, such as expression quantitative trait locus (eQTL) analysis or mediation analysis, as well as experimental validation, will be required to clarify the functional roles of the identified proteins. This pattern is consistent with the polygenic and multi-layer regulatory architecture of complex metabolic diseases.

This study suggests that MASLD may be more than a simple fat storage disorder; rather, it represents a network-based disease involving lipid metabolism, insulin/IGF signaling, mitochondrial function, and ECM–inflammatory responses. *PNPLA3* and *TM6SF2* mutations reaffirmed previously recognized major risk factors, while *SAMM50* and *NCAN* were highlighted for potential functional relevance through network-based analysis. Although the present study did not identify novel genome-wide significant loci, this may reflect the characteristics of the study population, which was restricted to a subset with available proteomic data. Importantly, the strength of this study lies in its integrative multi-omics approach combining genetic, proteomic, and network-level analyses. This framework provides a systems-level understanding of MASLD by linking established genetic variants to downstream molecular pathways and protein interaction networks. In this context, the findings extend beyond conventional GWAS by offering functional insights into how known risk loci may contribute to disease biology.

Moreover, proteins such as IGFBP2, ADIPOQ, LEP, and CPM emerged as candidates for diagnostic and prognostic biomarkers, as well as potential therapeutic targets. Collectively, these findings support a gene–protein–pathway model for MASLD pathophysiology and indicate that a network-based approach could aid in the development of early diagnostic tools and personalized therapeutic strategies. Nonetheless, further experimental and clinical validation will be required to substantiate these possibilities.

## 4. Materials and Methods

### 4.1. Study Population

This study utilized data from the UK Biobank, a large prospective cohort of approximately 500,000 individuals aged 40–69 years recruited between 2006 and 2010. Participants were selected from those with available hepatic magnetic resonance imaging-derived proton density fat fraction (PDFF), genetic data, and plasma proteomic data measured using the Olink platform. Hepatic steatosis was defined as an imaging-derived PDFF value of ≥5%, consistent with commonly used criteria for liver fat accumulation. To reflect the current MASLD framework, cases were defined as participants with PDFF ≥ 5% together with at least one metabolic abnormality, whereas controls were defined as participants with PDFF < 5% and without metabolic abnormalities. Metabolic abnormalities were defined using available clinical variables in the UK Biobank dataset. Because the present study was conducted within a population-based cohort using available imaging and proteomic data, alcohol intake and other liver diseases were not applied as strict exclusion criteria. Accordingly, the study population should be understood as an operationally defined cohort aligned with the MASLD framework within the UK Biobank (Manchester, UK).

### 4.2. Clinical and Biochemical Measures

Clinical data were extracted from the UK Biobank database, including anthropometric parameters, blood pressure, liver enzymes (AST, ALT, GGT), lipid profiles (LDL, HDL, TG), glucose, and inflammatory markers (CRP, neutrophils, lymphocytes, monocytes, NLR, CAR, PNI). These measures allowed comprehensive assessment of metabolic and immune status across MASLD and control groups.

### 4.3. Genome-Wide Association Analyses (GWAS)

Genome-wide association analyses (GWAS) were performed using the COMPASS platform (Cipherome Inc., San Jose, CA, USA) under additive, dominant, and recessive genetic models to capture potential non-additive genetic effects. The additive model was considered the primary analysis, whereas the dominant and recessive models were included as complementary approaches to explore alternative genetic architectures.

Quality control procedures implemented within the platform included filtering based on minor allele frequency (>0.01), Hardy–Weinberg equilibrium (*p* > 1 × 10^−6^), and genotype call rate (>95%), as well as restriction to autosomal variants and removal of related individuals. Covariate adjustment for factors such as age, sex, body mass index, and population structure (e.g., principal components) was not explicitly applied beyond the default settings of the analysis platform.

Manhattan and quantile–quantile (QQ) plots were generated using Python (v3.11.13) based on GWAS summary statistics exported from the COMPASS platform. Genomic inflation was assessed using QQ plots, as genomic inflation factors (λ) were not directly available from the platform. The QQ plots showed minimal deviation from the expected distribution, suggesting no substantial inflation of test statistics. Standard quality control procedures and the removal of related individuals were applied to reduce potential confounding; however, residual population stratification cannot be completely excluded.

Genome-wide significance was defined using a conventional threshold of *p* < 5 × 10^−8^ for each model. The additive model was considered the primary analysis, whereas the dominant and recessive models were used as complementary approaches to explore potential non-additive genetic effects. Given the exploratory nature of these non-additive models and the fact that the inheritance models are not independent, no additional multiple testing correction across models was applied. Accordingly, results from the dominant and recessive models were interpreted as supportive findings rather than primary evidence.

### 4.4. Functional Prediction and Conservation Analysis

To assess the functional consequences of significant variants, prediction scores were obtained from dbNSFP (v4.4) [50], including SIFT [51], PolyPhen-2 [52], PROVEAN [53], MutationTaster [54], REVEL [55], and CADD [56]. Deleteriousness thresholds followed standard cutoffs (SIFT < 0.05, PolyPhen-2 > 0.85, PROVEAN < −2.5, REVEL > 0.5, CADD ≥ 20). Evolutionary conservation was assessed using GERP++ [57], phyloP [58], and phastCons [59]. Human-specific constraint was evaluated using the B-statistic [16].

### 4.5. Plasma Proteomic Profiling

Plasma protein levels were analyzed using the Olink^®^ proximity extension assay platform (Olink Proteomics, Uppsala, Sweden). Data were expressed in normalized protein expression units on a log_2_ scale. Quality control was performed according to Olink’s standard operating procedures, with internal and external controls included. Differential expression between the MASLD and control groups was assessed using linear regression, adjusting for age and sex. Multiple testing correction was applied using the Benjamini–Hochberg (FDR < 0.05). Results were visualized in volcano plots and heatmaps.

### 4.6. PPI Network Analysis

Differentially expressed proteins (FDR < 0.05) were mapped into a PP network using STRING v12.0 [60] with a minimum required interaction score of 0.700. Nodes represent proteins and edges represent known or predicted interactions from curated databases, experimental data, and text mining. Clustering was performed using the MCL algorithm (inflation parameter = 2.0).

## Figures and Tables

**Table 1:** Demographic and clinical characteristics of participants.

	Normal (*n* = 3600) [Mean (SD)]	Case (*n* = 1008) [Mean (SD)]	*p* Value
Age	71.48 (7.54)	71.92 (7.29)	
Sex	Male (*n* = 1587) Female (*n* = 2013)	Male (*n* = 593) Female (*n* = 415)	
PDFF	1.97 (1.08)	10.84 (5.75)	<0.001
BMI	25.59 (4.51)	29.59 (4.79)	0.218
Glucose	89.04 (16.07)	93.93 (21.77)	0.038
HDL	59.13 (14.43)	50.13 (11.51)	0.107
TG	133.81 (69.95)	198.80 (101.39)	<0.001
DBP	79.21 (8.58)	83.34 (8.31)	0.23
SBP	134.45 (15.75)	140.82 (14.25)	0.827
AST	25.18 (8.90)	28.06 (9.32)	<0.001
ALT	20.93 (12.13)	29.85 (15.86)	<0.001
GGT	31.37 (29.35)	44.66 (41.41)	<0.001
CRP	1.92 (3.23)	2.86 (3.93)	<0.001
Neutrophil	4.10 (1.19)	4.41 (1.23)	0.041
Lymphocyte	1.85 (0.81)	2.05 (0.64)	0.001
Platelets	239.81 (52.62)	242.55 (54.97)	0.287
Monocytes	0.42 (0.15)	0.47 (0.17)	<0.001
Albumin	45.24 (2.43)	45.65 (2.38)	0.053
LDL	3.55 (0.80)	3.65 (0.86)	0.024
Lipoprotein	45.05 (49.04)	39.50 (46.49)	0.486
Protein	72.20 (3.77)	72.95 (3.64)	0.336
TyG index	4.63 (0.25)	4.85 (0.25)	<0.001
NLR	2.40 (1.00)	2.32 (1.01)	<0.001
PLR	140.19 (48.17)	127.25 (43.75)	0.988
PNI	45.25 (2.43)	45.66 (2.38)	0.047
NMR	10.82 (6.01)	10.52 (6.49)	0.459
CAR	0.06 (0.10)	0.05 (0.09)	0.001
AGR	1.71 (0.24)	1.70 (0.24)	0.177
DM	No 3480 (96.7%) Yes 120 (3.3%)	No 873 (86.6%) Yes 135 (13.4%)	0.079
HTN	No 2729 (75.8%) Yes 871 (24.2%)	No 593 (58.8%) Yes 415 (41.2%)	0.963
Hyperlipidemia	No 3442 (95.6%) Yes 158 (4.4%)	No 935 (92.8%) Yes 73 (7.2%)	0.247

**Table 2:** Genome-wide significant SNPs associated with proton density fat fraction (PDFF) across additive and dominant genetic models. The table presents SNPs significantly associated with hepatic PDFF identified through GWAS under genetic models: additive and dominant. For each SNP, the chromosome number, genomic position (GRCh37/hg19), reference and alternative alleles, alternative allele frequency, odds ratio (OR), standard error of the log-transformed OR [log(OR)_SE], Z statistic, and *p*-value are provided. Gene annotations and predicted functional consequences (e.g., coding variant, intron, intergenic) were derived using the Ensembl Variant Effect Predictor (VEP). SNPs reaching genome-wide significance (*p* < 5 × 10^−8^) are shown. Empty gene or location fields indicate variants located in intergenic regions.

Model	Chr	Position	ID	REF	ALT	ALT Freq	OR	LOG (OR)_SE	Z_STAT	*p*	Gene	Location
Additive	22	44324727	rs738409	C	G	0.214	1.558	0.059	7.569	3.76663 × 10^−14^	*PNPLA3*	Exon (I148M, coding variant)
Additive	19	19379549	rs58542926	C	T	0.076	1.919	0.086	7.545	4.50775 × 10^−14^	*TM6SF2*	Exon (E167K, coding variant)
Additive	19	19662220	rs17216525	C	T	0.082	1.742	0.083	6.673	2.50042 × 10^−11^	*TM6SF2* region	Likely intronic or regulatory
Additive	19	19658472	rs16996148	G	T	0.083	1.701	0.083	6.395	1.60753 × 10^−10^	*TM6SF2* region	Likely intronic or regulatory
Additive	19	19329924	rs2228603	C	T	0.076	1.728	0.088	6.242	4.3095 × 10^−10^	*TM6SF2* region	Likely intronic or regulatory
Additive	19	19789528	rs2304130	A	G	0.085	1.648	0.083	6.010	1.8571 × 10^−9^	*ZNF101*	Intron
Additive	22	44325996	rs12483959	G	A	0.154	1.476	0.066	5.908	3.45441 × 10^−9^	*PNPLA3*	Intron
Additive	22	44332570	rs2281135	G	A	0.160	1.446	0.065	5.678	1.3633 × 10^−8^	*PNPLA3*	Intron
Additive	22	44332888	Affx-19716376	T	TC	0.160	1.426	0.065	5.441	5.31307 × 10^−8^		
Dominant	22	44324727	rs738409	C	G	0.214	1.693	0.072	7.285	3.20712 × 10^−13^	*PNPLA3*	Exon 3 (I148M, coding variant)
Dominant	19	19379549	rs58542926	C	T	0.076	1.912	0.092	7.054	1.73962 × 10^−12^	*TM6SF2*	Exon (E167K, coding variant)
Dominant	22	44325996	rs12483959	G	A	0.154	1.591	0.076	6.100	1.06186 × 10^−9^	*PNPLA3*	Intron
Dominant	19	19662220	rs17216525	C	T	0.082	1.726	0.091	6.016	1.79011 × 10^−9^	*TM6SF2* region	Likely intronic or regulatory
Dominant	22	44332570	rs2281135	G	A	0.160	1.568	0.076	5.940	2.84285 × 10^−9^	*PNPLA3*	Intron
Dominant	19	19329924	rs2228603	C	T	0.076	1.733	0.093	5.909	3.45234 × 10^−9^	*TM6SF2* region	Likely intronic or regulatory
Dominant	22	44332888	Affx-19716376	T	TC	0.160	1.550	0.076	5.774	7.73232 × 10^−9^		
Dominant	19	19658472	rs16996148	G	T	0.083	1.685	0.091	5.751	8.84622 × 10^−9^	*TM6SF2* region	Likely intronic or regulatory
Dominant	22	44368122	rs3761472	A	G	0.151	1.520	0.077	5.448	5.10198 × 10^−8^	*SAMM50*	Exon (A110G, coding variant)
Dominant	19	19789528	rs2304130	A	G	0.085	1.621	0.090	5.373	7.7651 × 10^−8^	*ZNF101*	Intron

**Table 3:** In silico functional prediction of MASLD-associated variants.

SNP (Gene)	Amino Acid Change	SIFT	PolyPhen-2	MutationTaster	PROVEAN	REVEL	CADD
rs58542926 (*TM6SF2*, E167K)	Glu → Lys	Tolerated (0.296)	Damaging (0.996)	Disease causing	Neutral (−1.19)	0.075	24.6
rs2228603 (*NCAN*, P → S)	Pro → Ser	Tolerated (0.225)	Damaging (0.957)	Possibly damaging	Neutral (−2.04)	0.098	21.3
rs738409 (*PNPLA3* I148M)	Ile → Met	Damaging (0.054)	Damaging (0.994)	Disease causing	Damaging (−2.57)	0.339	13.4
rs3761472 (*SAMM50* D → G)	Asp → Gly	Tolerated (0.213)	Benign (0.010)	Polymorphism	Neutral (−1.24)	0.080	16.7

**Table 4:** Conservation analysis of MASLD-associated variants.

Variant	Position	GERP++ RS	phyloP	phastCons	B-Statistic
rs738409 *PNPLA3* I148M	22:44324727	−1.97 (low)	−1.142 (low)	0.009 (very low)	855 (high)
rs58542926 *TM6SF2* E167K	19:19379549	5.01 (high)	3.459 (high)	0.996 (very high)	794 (medium)
rs3761472 *SAMM50* D → G	22:44368122	4.78 (high)	3.658 (high)	0.998 (very high)	798 (medium)
rs2228603 *NCAN* P → T	19:19329924	4.66 (high)	1.689 (medium)	0.985 (very high)	740 (medium)

**Table 5:** Top 5 up- and downregulated differentially expressed plasma proteins associated with proton density fat fraction.

Protein (Full Name)	Normal Mean (Std)	Case Mean (Std)	Unadjusted *p*-Value	Coefficient	Adjusted *p*-Value
IGFBP2 (insulin-like growth factor binding protein 2)	0.054 (0.732)	−0.575 (0.755)	1.17 × 10^−17^	−0.612	3.78 × 10^−19^
IGFBP1 (insulin-like growth factor binding protein 1)	0.15 (1.358)	−0.987 (1.446)	2.45 × 10^−16^	−1.055	8.44 × 10^−17^
PON3 (paraoxonase 3)	0.048 (0.293)	−0.199 (0.4)	1.30 × 10^−11^	−0.213	3.40 × 10^−13^
CKB (creatine kinase B-type)	0.064 (0.646)	−0.413 (0.677)	1.40 × 10^−10^	−0.473	7.23 × 10^−12^
APOF (apolipoprotein F)	0.016 (0.252)	−0.174 (0.272)	1.62 × 10^−10^	−0.173	1.72 × 10^−10^
CPM (carboxypeptidase M)	−0.131 (0.42)	0.248 (0.379)	6.86 × 10^−23^	0.336	4.65 × 10^−19^
IGSF9 (immunoglobulin superfamily member 9)	−0.256 (0.782)	0.561 (0.877)	1.82 × 10^−16^	0.770	1.25 × 10^−18^
GUSB (beta-glucuronidase)	−0.214 (0.662)	0.404 (0.732)	3.01 × 10^−18^	0.523	3.78 × 10^−18^
ACY1 (aminoacylase 1)	−0.184 (0.702)	0.471 (0.797)	8.51 × 10^−18^	0.557	8.46 × 10^−18^
AFM (afamin)	−0.069 (0.198)	0.122 (0.205)	1.55 × 10^−16^	0.183	2.82 × 10^−17^

**Table 6:** STRING MCL cluster descriptions.

Cluster Number	Gene Count	Primary Description	Secondary Description	Protein Names
1	16	Response to cAMP	-	IGFBP1, OXT, REN, LEP, SHBG, PLAT, LPL, SELE, SERPINF1, IGFBP2, ADIPOQ, FGF21, GHR, FABP4, GPD1, RARRES2
2	9	-	-	SEMA3F, MET, CDH2, HGF, KRT18, ERBB2, ITGA5, DSG2, CDH15
3	5	Serine-type endopeptidase complex	Complement cascade	F9, APCS, AFM, CFH, CFI
4	5	-	-	KYNU, FTCD, UPB1, ASGR1, ACY1
5	4	Drug metabolism-cytochrome P450	-	GSTA3, ADH4, ADH1B, GSTA1
6	4	Glycoside catabolism	Metabolism of Angiotensinogen to Angiotensins	GLA, CTSD, GUSB, CES1
7	4	LDL	Low-density lipoprotein particle	APOF, PON3, LDLR, SCLY
8	3	-	-	BPIFB2, FAM20A, VWA1
9	3	Formation of xylulose-5-phosphate	Fructose and mannose metabolism	SORD, FBP1, DCXR
10	2	-	-	NPL, PRAP1
11	2	Interleukin-1 receptor activity, and Interleukin-1 family	-	IL1RN, IL18R1
12	2	-	-	ATRN, CA14
13	2	Tetrahydrobiopterin biosynthesis	-	PTS, PCBD1
14	2	Carboxypeptidase	-	PRCP, CPM
15	2	-	-	FURIN, ADAMTS15

## Data Availability

The data analyzed in this study are available from the UK Biobank upon application and approval. Restrictions apply to the availability of these data, which were used under license for the current study and are therefore not publicly available.

## Abbreviations

AGT, albumin-to-globulin ratio; ALT, alanine aminotransferase; AST, aspartate aminotransferase; BMI, body mass index; CAR, CRP-to-albumin ratio; CRP, C-reactive protein; DBP, diastolic blood pressure; DM, diabetes mellitus; FDR, false discovery rate; GGT, γ-glutamyl transferase; GWAS, genome-wide association studies; HDL, high-density lipoprotein; HTN, hypertension; LDL, low-density lipoprotein; MASLD, metabolic dysfunction-associated steatotic liver disease; NAFLD, non-alcoholic fatty liver disease; NLR, neutrophil-to-lymphocyte ratio; NMR, neutrophil-to-monocyte ratio; PDFF, proton density fat fraction; PLR, platelet-to-lymphocyte ratio; PNI, prognostic nutritional index; PPI, protein–protein interaction; SBP, systolic blood pressure; SNP, single nucleotide polymorphism; TG, triglycerides; TyG, triglyceride-glucose.
